# Group B Streptococcus-Associated Necrotizing Soft Tissue Infection Following Minor Ankle Trauma: A Case Report

**DOI:** 10.7759/cureus.115444

**Published:** 2026-08-30

**Authors:** Allen Sangar, Martin L Molina, Andrew Sangar, Ashley Falk

**Affiliations:** 1 Transitional Year, Florida State University College of Medicine, Winter Haven, USA; 2 Family Medicine, Florida State University College of Medicine, Winter Haven, USA; 3 School of Medicine, Loma Linda University Medical Center, Loma Linda, USA

**Keywords:** diabetes mellitus, diabetic ketoacidosis, group b streptococcus, necrotizing fasciitis, necrotizing soft tissue infection, streptococcus agalactiae, transtibial amputation

## Abstract

Necrotizing soft tissue infections (NSTIs) are rapidly progressive, life- and limb-threatening emergencies requiring immediate surgical source control. Group B Streptococcus (GBS; *Streptococcus agalactiae*) is a rare cause of NSTIs in adults.

We present a 44-year-old woman with poorly controlled diabetes who developed progressive right foot pain and swelling after minor ankle trauma. Examination showed spreading erythema, crepitus, dusky discoloration, malodor, and a deep plantar wound, accompanied by marked leukocytosis and diabetic ketoacidosis. Computed tomography demonstrated infection extending from the forefoot through the ankle into the anterior compartment of the leg, prompting emergent exploration that confirmed extensive NSTI. Two wound specimens obtained before antibiotics yielded heavy GBS growth, whereas deep intraoperative ankle and leg specimens collected after starting antibiotics grew heavy *Streptococcus anginosus* and moderate *Prevotella* species. Despite broad-spectrum antibiotics and debridement, transtibial amputation was required.

This case highlights a rare GBS-associated polymicrobial NSTI and emphasizes early recognition of progressive soft-tissue findings despite an apparently minor musculoskeletal injury.

## Introduction

Necrotizing soft tissue infections (NSTIs) are rapidly progressive infections involving the subcutaneous tissues, fascia, and sometimes muscle. The estimated incidence in the United States is 3.8-10.3 cases per 100,000 people annually, with an in-hospital mortality of 10%-20% [1-3]. NSTIs are classified as type I (polymicrobial) or type II (monomicrobial), with group A Streptococcus and *Staphylococcus aureus* being the most common monomicrobial pathogens [1,3,4]. Early manifestations can be nonspecific, but progression may cause multiorgan failure, limb loss, and death. Diabetes mellitus is a major predisposing condition and is present in approximately 45% of cases [5]. Immediate surgical evaluation, broad empiric antimicrobial therapy, and prompt debridement are central to management [1,3-5].

Group B Streptococcus (GBS; *Streptococcus agalactiae*) is increasingly recognized as a cause of serious infection in nonpregnant adults, particularly those with diabetes and other chronic conditions; however, GBS-associated necrotizing infection remains rare [6-9]. A 2016 review identified 22 cases of GBS necrotizing fasciitis in nonpregnant adults reported in the English-language literature; most were monomicrobial, 73% occurred in patients with diabetes, and 70% involved the lower extremities. Only three of those 22 cases were polymicrobial [6]. The overall frequency of polymicrobial NSTI involving GBS remains unclear.

*Streptococcus anginosus* is an abscess-forming organism that is associated with pyogenic and polymicrobial soft-tissue infections [10,11]. Gas-forming myonecrosis attributed to this organism has rarely been described [12]. In a clinical series of 332 *S. anginosus* group infections, 72% involved skin and soft tissue and 70% were polymicrobial, but necrotizing fasciitis was not specifically quantified as a subset [11]. *Prevotella* species are obligate anaerobic gram-negative commensals of mucosal surfaces. They may act as opportunistic pathogens in devitalized tissue and are recognized components of type I (polymicrobial) NSTI [13-15]. 

We report a rare GBS-associated polymicrobial NSTI involving *S. anginosus* and *Prevotella* in a woman with poorly controlled diabetes. The infection presented after minor ankle trauma, progressed proximally through the lower extremity, and ultimately required transtibial amputation and subsequent rehabilitation.

## Case presentation

A 44-year-old woman with poorly controlled diabetes mellitus and inconsistent medication use was transported to the Emergency Department for progressive right foot pain and swelling. Several days earlier, she had sustained a minor twisting injury to the ankle but initially remained ambulatory. Over the following days, her symptoms worsened, with increasing pain, swelling, calf tenderness, nausea, and subjective fever, until the pain became severe enough to limit weight-bearing.

On presentation, she was afebrile at 98°F (36.7°C) but tachycardic at 106 beats/min. Examination revealed a markedly warm, malodorous right foot with erythema extending above the ankle, dorsal crepitus, dusky skin discoloration, and a deep plantar wound beneath the first metatarsal head.

Laboratory findings at presentation are summarized in Table 1. The results demonstrated leukocytosis, diabetic ketoacidosis, and elevated inflammatory markers.

**Table 1:** Laboratory findings at presentation.

Laboratory test	Result	Units	Reference range
White blood cell count	33.4	×10³/µL	4.5-11.0
Glucose	371	mg/dL	70-99
Bicarbonate	14	mmol/L	21-32
Anion gap	18	-	5-16
Beta-hydroxybutyrate	4.41	mmol/L	0-0.70
Hemoglobin A1c	13.2	%	4.7-5.6
C-reactive protein	38.24	mg/dL	0.00-0.50
Erythrocyte sedimentation rate	126	mm/h	0-20
Lactate	1.7	mmol/L	0.4-2.0
Procalcitonin	3.76	ng/mL	0.00-0.24
Creatinine	0.63	mg/dL	0.50-1.10

Before antimicrobial therapy, blood cultures and wound specimens from a deep skin abscess and toe ulcer were obtained. Piperacillin-tazobactam 4.5 g IV and vancomycin 2 g IV were then administered. Intravenous fluids and an insulin infusion were initiated for sepsis and diabetic ketoacidosis.

Computed tomography (CT) demonstrated a plantar forefoot ulcer with extensive soft-tissue gas extending through the foot and ankle and proximally within the anterior compartment of the leg to just below the knee (Figures 1-2). No definitive evidence of acute osteomyelitis was identified. Because of sepsis, diabetic ketoacidosis, and concern for rapidly progressive necrotizing infection, the patient was transferred to the operating room for emergent incision, drainage, and excisional debridement on hospital day 1.

**Figure 1:**
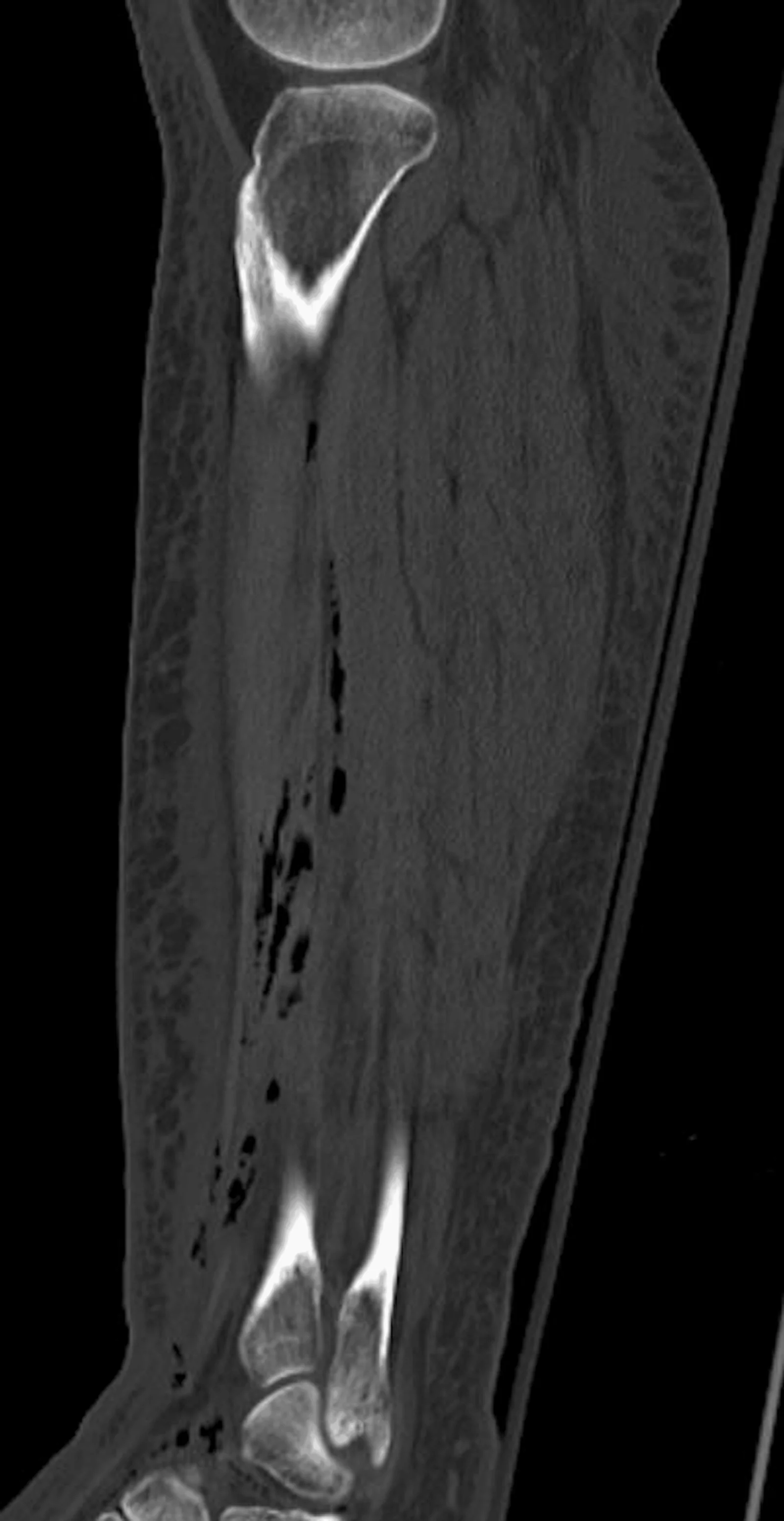
Sagittal computed tomography image of the right lower extremity demonstrating soft-tissue gas extending proximally within the anterior compartment of the leg.

**Figure 2:**
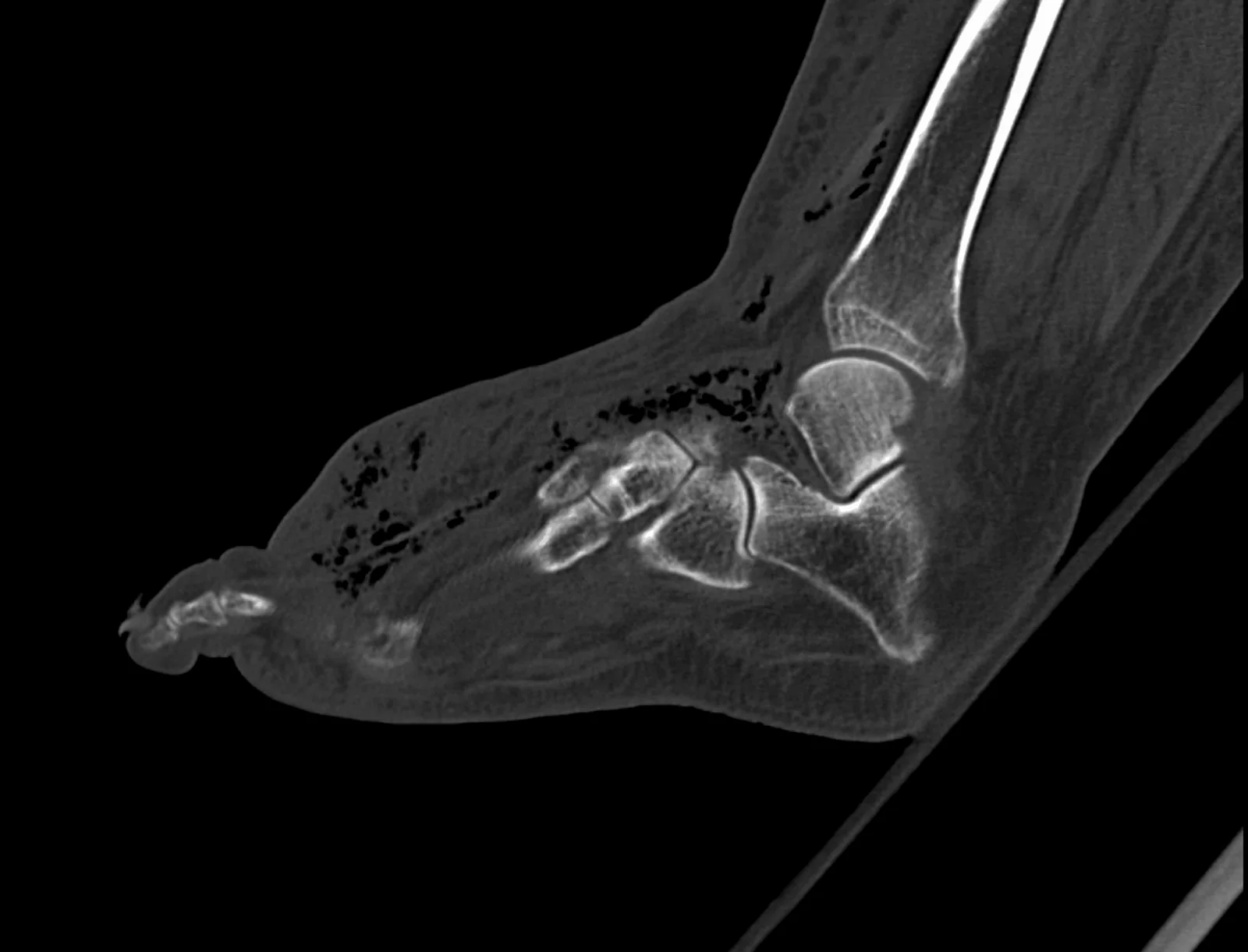
Sagittal computed tomography image of the right foot and ankle demonstrating extensive soft-tissue emphysema along the dorsum of the foot with extension toward the anterior ankle.

Surgical exploration revealed extensive malodorous purulence and myonecrosis involving the dorsal foot, anterior ankle, and anterior compartment of the lower leg. Necrosis involved soft tissue, fascia, muscle, and tendon, including the extensor hallucis longus musculotendinous unit, with proximal extension through the distal leg. Extensive debridement was performed, and the wound was left open. Given the degree of tissue destruction and poor likelihood of limb salvage, the operating surgeon documented a high probability of major amputation. Intraoperative deep abscess specimens were obtained from the right ankle and leg approximately five hours after the initial antimicrobial doses. Clindamycin 900 mg IV was administered shortly after specimen collection, and additional doses of piperacillin-tazobactam and vancomycin were given later that evening. The patient remained intubated postoperatively and was transferred to the Intensive Care Unit. The extensive postoperative wound following initial debridement is shown in Figure 3.

**Figure 3:**
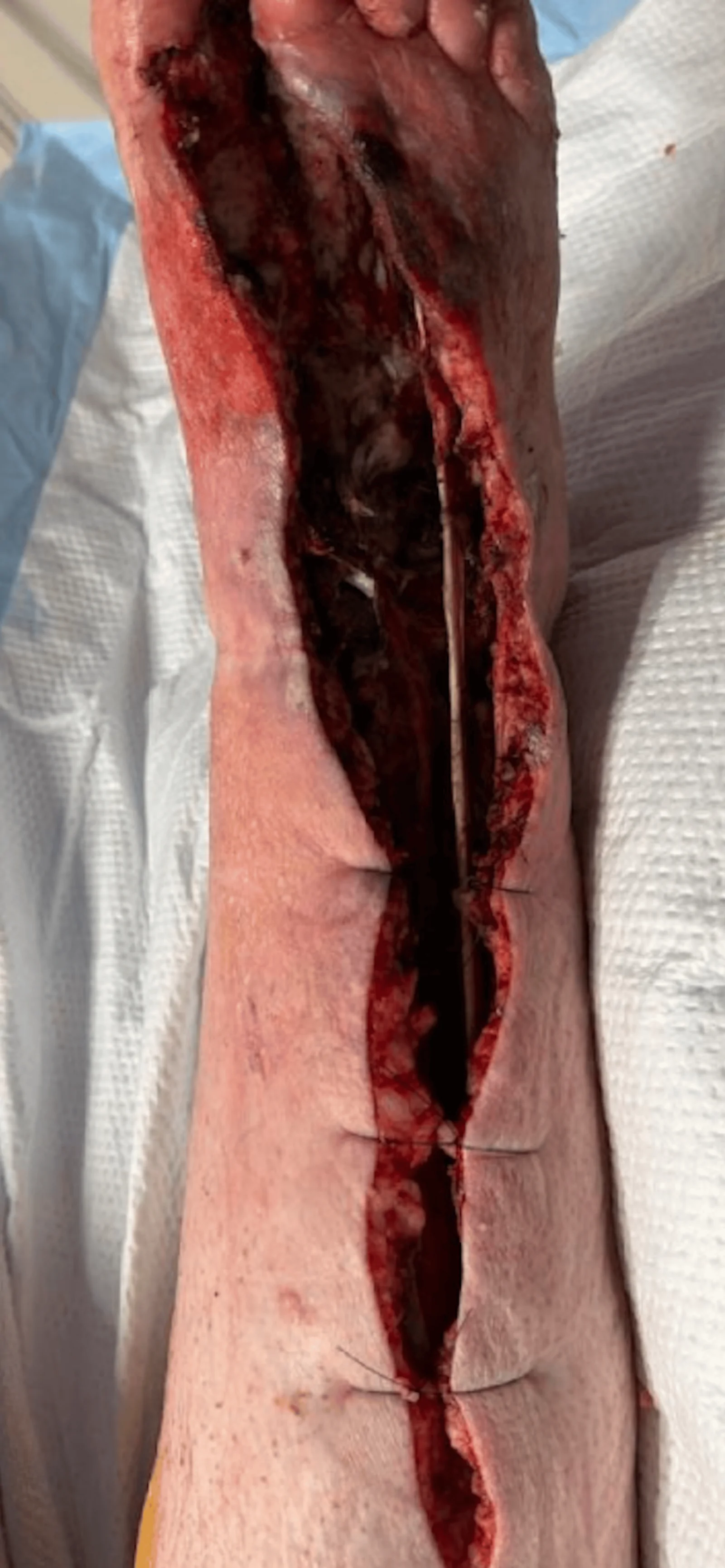
Right lower extremity on postoperative day 1 after extensive incision, drainage, and debridement.

Blood cultures showed no growth. The pre-antibiotic deep skin abscess and toe-ulcer specimens both yielded heavy GBS within 24 hours. The intraoperative right ankle and leg specimens subsequently grew heavy *S. anginosus* and moderate *Prevotella *species. Collectively, these findings supported a GBS-associated polymicrobial NSTI.

Despite aggressive debridement and broad-spectrum antimicrobial therapy, she remained in septic shock. On hospital day 2, she underwent an open right below-the-knee amputation for definitive source control. Approximately six days later, the residual limb was revised and closed.

Her postoperative course was complicated by anuric acute kidney injury, with creatinine increasing from 0.63 to approximately 2.8 mg/dL. Temporary hemodialysis was initiated for presumed ischemic acute tubular necrosis in the setting of sepsis, and renal function eventually recovered.

Following amputation and critical illness, she participated in physical therapy focused on strengthening, transfer training, and wheelchair mobility. She progressed to independent wheelchair transfers and was discharged with a wheelchair after a prolonged hospitalization complicated by disposition barriers.

## Discussion

Microbiology is the principal distinguishing feature of this case. NSTIs are commonly polymicrobial or associated with group A Streptococcus. Although GBS is classically associated with maternal and neonatal infections, it is an uncommon cause of NSTI in nonpregnant adults. Reported cases often occur in patients with diabetes, malignancy, or other chronic illnesses [6-9]. In this patient, a hemoglobin A1c of 13.2% and concurrent diabetic ketoacidosis reflected substantial chronic and acute metabolic dysregulation, likely increasing susceptibility to severe infection. 

The culture chronology supported a GBS-associated polymicrobial NSTI. A deep skin abscess specimen and a toe-ulcer specimen obtained before antibiotic administration both yielded heavy GBS growth, supporting its clinical significance rather than incidental colonization. Deep abscess specimens collected intraoperatively from the right ankle and leg approximately five hours after piperacillin-tazobactam and vancomycin were initiated subsequently grew heavy *S. anginosus* and moderate *Prevotella* species. 

*S. anginosus* is recognized for its association with abscess formation and invasive purulent infection, frequently in polymicrobial settings. Although *S. anginosus* may be isolated from diabetic foot infections, its association with myonecrosis, soft-tissue gas, and necrotizing fasciitis is uncommon and has largely been described in case reports [10-12]. *Prevotella* is a plausible companion organism in devitalized, anaerobic tissue and is recognized in polymicrobial NSTI and diabetes-related foot infections [13-15]. The combination of heavy GBS in two pre-antibiotic specimens and heavy *S. anginosus* with *Prevotella* in deep operative specimens therefore represents an unusual microbiologic pattern. Prior antibiotic exposure may have affected later culture recovery, but the direction and magnitude of this effect cannot be determined retrospectively [16]. The infection is consequently best classified as a GBS-associated polymicrobial NSTI rather than monomicrobial or definitively GBS-predominant disease.

The preceding ankle twist provided a plausible musculoskeletal explanation for the initial pain and swelling but was not established as the cause of infection. The plantar ulcer may have served as the portal of entry, although its temporal relationship to the injury was unclear (Figure 4). Progressive pain, inability to bear weight, spreading erythema, dusky discoloration, malodor, a deep wound, and crepitus were inconsistent with an uncomplicated ankle injury. This presentation emphasizes the importance of removing shoes and dressings and performing a complete skin, plantar-surface, soft-tissue, neurologic, and vascular examination in patients with diabetes who present with lower-extremity pain.

**Figure 4:**
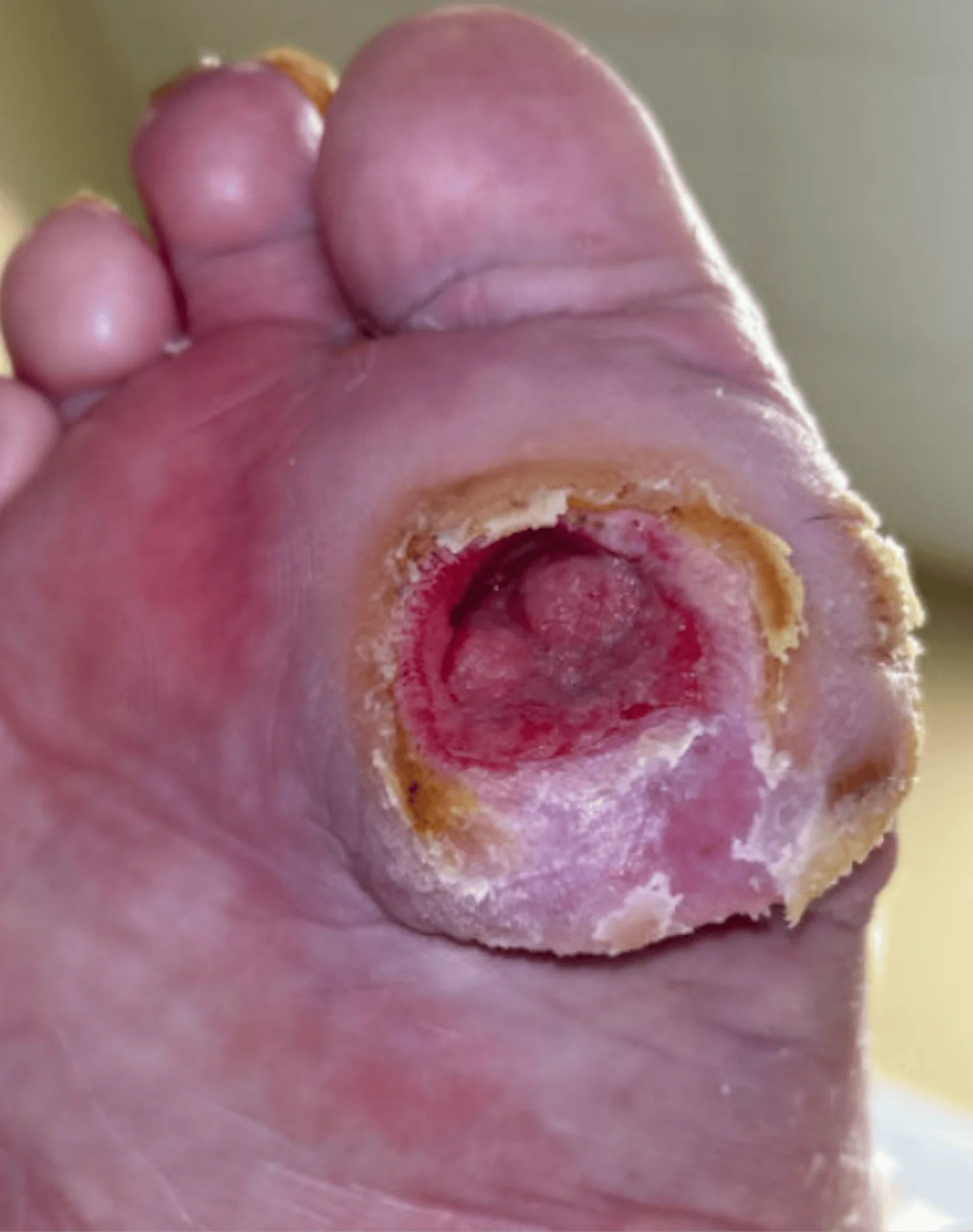
Right plantar ulcer beneath the first metatarsal head at presentation.

The absence of fever and a lactate of 1.7 mmol/L did not reflect the anatomic severity of the infection. Her rapidly progressive symptoms, marked leukocytosis, diabetic ketoacidosis, abnormal soft-tissue findings, and extensive gas on CT were more informative than any isolated vital sign or laboratory value. Imaging delineated the proximal extent of disease, whereas surgical exploration confirmed necrosis involving fascia, muscle, tendon, and other soft tissues. Imaging should not delay operative exploration when clinical suspicion for NSTI is high [1,3,4].

The consequences extended beyond infection control. The patient developed anuric acute kidney injury requiring temporary hemodialysis, underwent repeat surgery, and experienced substantial deconditioning. Following amputation, physical therapy and wheelchair provision enabled her to achieve independent wheelchair transfers before discharge. This functional improvement underscores the importance of early rehabilitation addressing mobility, equipment needs, residual-limb care, contracture prevention, psychological adjustment, and future prosthetic candidacy.

## Conclusions

GBS is a rare cause of adult NSTI but may contribute to severe polymicrobial infection, particularly in patients with poorly controlled diabetes. In this patient, rapidly progressive foot infection after minor ankle trauma extended proximally through the leg and ultimately required transtibial amputation.

Progressive pain, impaired weight-bearing, deep wounds, malodor, spreading erythema, skin discoloration, and crepitus should prompt urgent evaluation for NSTI even when symptoms initially appear musculoskeletal and the patient is afebrile. Prompt surgical source control, broad-spectrum antimicrobial therapy, and deep-tissue cultures are essential. For patients requiring major amputation, early rehabilitation is also critical for restoring mobility and functional independence.
